# Dr. Augustus Currie

**Published:** 1911-04

**Authors:** 


Dr. Augustus Currie, a graduate of the University of Buffalo (1864) died at his home in Englewood, N. J. February 28, 1911, aged 70 years.

